# The N-terminal domain of human mitochondrial helicase Twinkle has DNA-binding activity crucial for supporting processive DNA synthesis by polymerase γ

**DOI:** 10.1016/j.jbc.2022.102797

**Published:** 2022-12-14

**Authors:** Laura C. Johnson, Anupam Singh, Smita S. Patel

**Affiliations:** 1 Department of Biochemistry and Molecular Biology, Robert Wood Johnson Medical School, Rutgers University, Piscataway, New Jersey, USA; 2 Graduate School of Biomedical Sciences, Robert Wood Johnson Medical School, Rutgers University, Piscataway, New Jersey, USA

**Keywords:** mitochondria, replication, helicase, Twinkle, DNA polymerase, replisome, mitochondrial diseases, CTD, C-terminal domain, FL, full length, HP, high performance, mt, mitochondrial, mtDNA, mitochondrial DNA, Ni, nickel, NTD, N-terminal domain, PEI, polyethyleneimine, Polγ, DNA polymerase γ, TCEP, Tris(2-carboxyethyl)phosphine

## Abstract

Twinkle is the ring-shaped replicative helicase within the human mitochondria with high homology to bacteriophage T7 gp4 helicase–primase. Unlike many orthologs of Twinkle, the N-terminal domain (NTD) of human Twinkle has lost its primase activity through evolutionarily acquired mutations. The NTD has no demonstrated activity thus far; its role has remained unclear. Here, we biochemically characterize the isolated NTD and C-terminal domain (CTD) with linker to decipher their contributions to full-length Twinkle activities. This novel CTD construct hydrolyzes ATP, has weak DNA unwinding activity, and assists DNA polymerase γ (Polγ)-catalyzed strand-displacement synthesis on short replication forks. However, CTD fails to promote multikilobase length product formation by Polγ in rolling-circle DNA synthesis. Thus, CTD retains all the motor functions but struggles to implement them for processive translocation. We show that NTD has DNA-binding activity, and its presence stabilizes Twinkle oligomerization. CTD oligomerizes on its own, but the loss of NTD results in heterogeneously sized oligomeric species. The CTD also exhibits weaker and salt-sensitive DNA binding compared with full-length Twinkle. Based on these results, we propose that NTD directly contributes to DNA binding and holds the DNA in place behind the central channel of the CTD like a “doorstop,” preventing helicase slippages and sustaining processive unwinding. Consistent with this model, mitochondrial single-stranded DNA-binding protein (mtSSB) compensate for the NTD loss and partially restore kilobase length DNA synthesis by CTD and Polγ. The implications of our studies are foundational for understanding the mechanisms of disease-causing Twinkle mutants that lie in the NTD.

Twinkle is the replicative helicase of the human mitochondria, discovered through its sequence homology to bacteriophage T7 gp4 helicase–primase in linkage studies of mitochondrial-related disease, autosomal dominant progressive external ophthalmoplegia (1). Many point mutations of Twinkle have been identified in association with clinical presentations of a number of varied mitochondrial-related neuromuscular diseases, from the aforementioned autosomal dominant progressive external ophthalmoplegia to mitochondrial DNA (mtDNA) depletion syndrome, spinocerebellar ataxia, and certain presentations of Parkinson’s symptoms (2, 3, 4, 5). These mutations disturb mtDNA replication and generally result in age-related mtDNA deletions or depletion (6). The expression of recombinant human Twinkle in bacteria and insect cells has greatly facilitated biochemical and structural studies of Twinkle and led to reconstituted mt replisome complex with the partnering proteins, the mtDNA polymerase γ (Polγ), which is a heterotrimer of PolγA and two PolγB subunits, and the mitochondrial single-stranded binding proteins (mtSSB) (7, 8). Twinkle spontaneously organizes into ring-shaped oligomers (7, 9). A recent cryo-EM study reported a high-resolution structure of a disease-mutant of Twinkle in a closed heptameric/octameric ring arrangement (Fig. 1) (10). Biochemical studies indicate that Twinkle is a 5′-3′ helicase with poor unwinding activity on its own (7, 8, 11). However, in concert with Polγ and mtSSB, Twinkle supports creation of kilobase-sized products in rolling circle DNA synthesis on a minicircle fork (8). Twinkle also demonstrates DNA annealing activity, the biological role of which remains unknown (7, 12).

**Figure 1:**
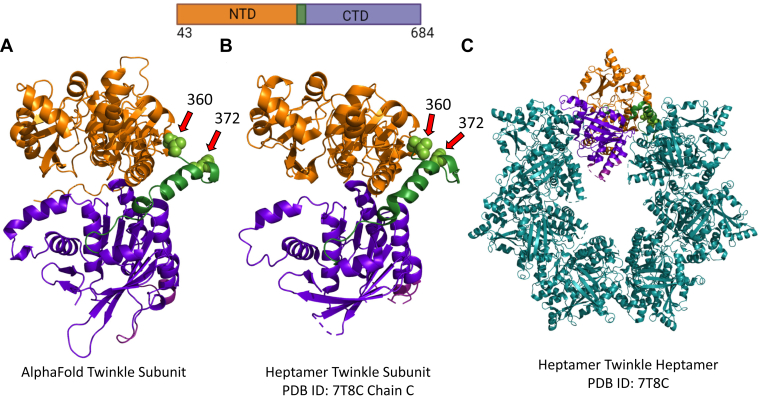
**Domain structure of the Twinkle subunit and its organization into a ring.***A*, the AlphaFold predicted structure for human Twinkle with the NTD in *orange*, the CTD in *purple*, and the linker region in *green*. Deletion position residues 360 and 372 are rendered as *spheres* and marked with *arrows*. *B*, the isolated subunit of the heptameric Twinkle (Protein Data Bank ID: 7T8C). *C*, cryo-EM structure of the heptameric Twinkle ring (Protein Data Bank ID: 7T8C). The primary structure schematic of the full-length construct with included amino acid residues is displayed above with the domains color coded. CTD, C-terminal domain; NTD, N-terminal domain.

Like bacteriophage T7 gp4 helicase–primase (13), each subunit of Twinkle consists of two roughly equivalently sized globular domain halves divided into an N-terminal domain (NTD) and a C-terminal domain (CTD), separated by a helical linker (Fig. 1). The CTD contains the conserved helicase and ATPase motifs (14) and the helix bundle that along with the linker organizes subunit–subunit interactions in ring assembly (10). The NTD includes primase motifs, and while many orthologs of the human Twinkle have retained the primase function (15, 16, 17) originating from T7 gp4 helicase–primase (18, 19, 20, 21), human Twinkle has lost this function because of mutations in the zinc-binding domain and Mg ion–binding site (14, 15, 16, 22). The role of replicative primase in human mitochondria is delegated to the mitochondrial RNA polymerase, POLRMT (23, 24). Unlike CTD, the NTD does not participate in intersubunit interactions but shows many intramolecular interactions with the CTD (10) (Fig. S1). A previous study reported biochemical analyses of a partially deleted NTD mutant of Twinkle (314–684 amino acids) and suggested that NTD is essential for replication functions (25). However, this construct retained much of the NTD proximal to the linker, which may be involved in intramolecular contacts (Fig. S1) (10). Furthermore, as no activity has been demonstrated for the NTD itself, its role in human Twinkle has remained mysterious. The NTD and linker are hot spots for disease-causing mutations (26), suggesting their vital role in mtDNA replication. Deciphering the NTD function is critical for understanding the mechanism of mutation-related replication defects.

In this study, we have expressed the isolated domains of human Twinkle protein (*TWNK*), including the NTD and CTD plus linker, referred to as the CTD here, and carried out a detailed biochemical analyses of these constructs and the full-length (FL) Twinkle, comparing their oligomerization, DNA binding, ATPase, helicase, and replisome functions. Our studies reveal that NTD has a DNA-binding activity, and although CTD can bind DNA on its own, the NTD confers high-affinity binding to FL Twinkle. In addition, our studies also show that NTD is required to form homogenous oligomers of Twinkle. Both DNA binding and proper oligomerization of Twinkle are essential for processive unwinding to facilitate production of multikilobase lengths of DNA through strand-displacement DNA synthesis by Polγ. We propose a doorstop model to explain the role of NTD in processive DNA unwinding.

## Results

### Expression of NTD and CTD protein domains of human Twinkle

To create the isolated domains of Twinkle, we used the AlphaFold predicted structure, which matches the recently determined cryo-EM structure of a human Twinkle mutant (10, 27). The experimental and predicted structure of the Twinkle subunit show that the NTD ends around amino acid residue 360, the linker helix region lies between 360 and 394, and the CTD is formed between 394 and 684 (Fig. 1). Accordingly, we cloned the sequence corresponding to amino acid residues 43 to 372 to make the NTD construct (35,596 Da) lacking the linker domain; the 1 to 42 amino acids is the predicted mitochondrial targeting sequence. This NTD construct was reported in a previously published study of Twinkle (25), whereas the minimal CTD construct (36,650 Da) that contained amino acids from 360 to 684, which includes the CTD and the linker region only, is novel. The SUMO-fusion constructs of NTD, CTD, and FL Twinkle were expressed in bacteria and purified as soluble proteins without the SUMO tag. The NTD was expressed abundantly compared with the CTD and FL Twinkle. We have reported the bacterial expression and purification of wildtype FL Twinkle previously (12), but the modified protocol described here produces soluble Twinkle without contaminating nucleic acid, chaperone, or exonuclease activity (Fig. S2).

### Size-exclusion gel filtration analysis of CTD, NTD, and FL Twinkle

**Figure 2:**
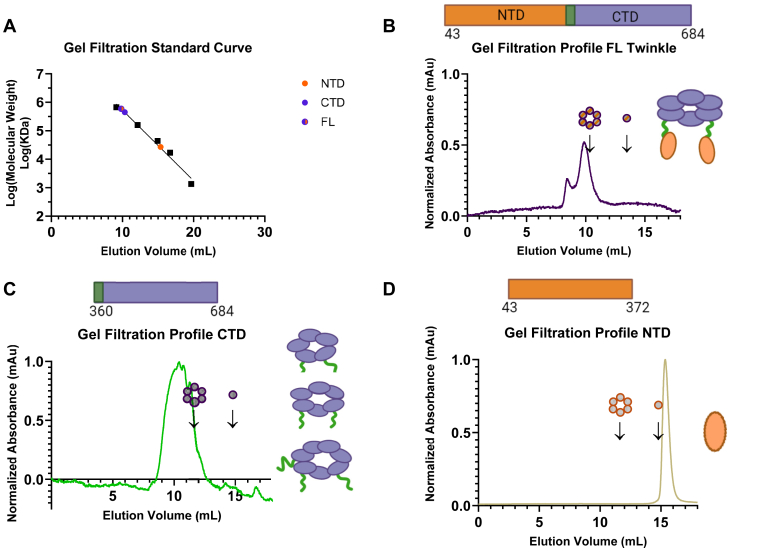
**Gel-filtration analysis to assess the oligomerization of purified FL Twinkle, CTD, and NTD constructs.** Samples were injected in Superdex 200 Increase 10/300 GL Cytiva column with respective running at 0.3 ml/min and peak elution volume monitored *via* 280 nm absorbance. *A*, calibration curve shows the elution volumes of the Bio-Rad gel filtration protein standards as *black squares*, and the elution volume of each Twinkle construct is displayed as *appropriately colored circles*. *B*, FL human Twinkle elution profile monitored using absorbance at 280 nm. Expected locations of hexameric and monomeric FL Twinkle are marked with *arrows* as determined by the calibration curve, major peak elutes ∼heptamer. *C*, Twinkle CTD elution profile with the expected locations of hexameric and monomeric CTD marked with *arrows*, peak elution correlates to a dodecamer. *D*, Twinkle NTD elution profile with the expected locations of hexameric and monomeric NTD marked with *arrows*, peak elution corresponds to a monomer. The oligomerization state(s) most likely present in each spectrum based on the elution profile is displayed to the *right*, whereas the primary structure schematic of each construct with included amino acid residues is displayed above each respective graph. CTD, C-terminal domain; FL, full length; NTD, N-terminal domain.

Twinkle belongs to the SF4 family of helicases, which are actively functional in a ring-shaped hexameric form (13, 28). Previous studies of Twinkle have observed FL Twinkle as either a hexamer or a heptamer with supporting evidence and corroborating observations for both cases in various conditions (9, 10, 29). We used size-exclusion gel filtration chromatography to compare and gauge construct assembly fitness (Fig. 2). Although limitations in gel filtration resolution do not allow for the determination of a single precise oligomeric state, low molecular weight conformers corresponding to one to three subunits of FL Twinkle can be distinguished from one another and form larger order oligomers corresponding to 5+ subunits of FL Twinkle. In the elution spectra of FL Twinkle, CTD, and NTD, we mark the expected elution volumes of a hexamer and a monomer based on the elution volumes of the protein markers (Fig. 2*A*). The FL Twinkle elution peak is close to a heptamer, and there was a small peak corresponding to protein aggregates (Fig. 2*B*). The CTD elution peak was broader and centered around a larger oligomeric species (∼12 subunit) (Fig. 2*C*). The NTD eluted as a monomer (Fig. 2*D*), even at the high concentrations of NTD used in this experiment (∼80 μM). These results indicate that CTD can oligomerize independently, but the broader elution peak of CTD relative to FL Twinkle indicates that the NTD domains regulate the oligomeric structure of Twinkle, preventing higher order species with likely poor functionality, and favoring competent ring formation.

### FL Twinkle and CTD bind ssDNA in a length-dependent manner

**Figure 3:**
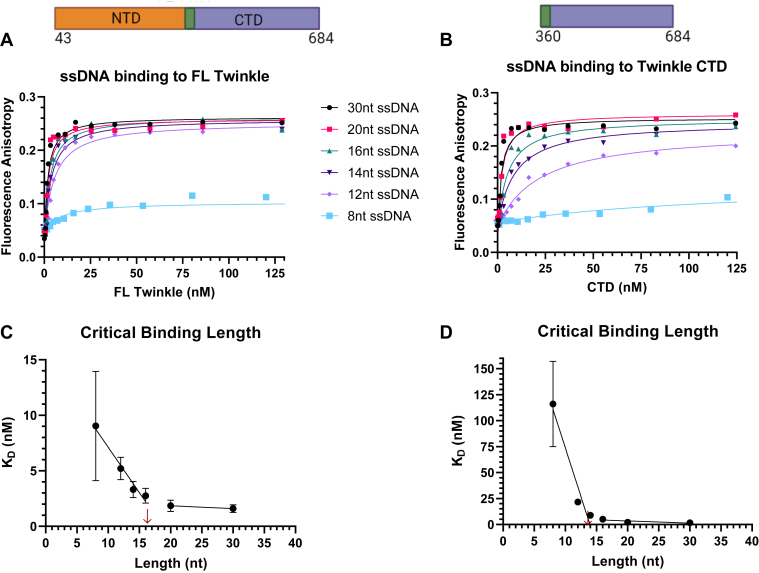
**Length-dependent binding of ssDNAs to FL Twinkle and CTD.** The fluorescence anisotropy–based titrations were carried out in buffer containing 50 mM Tris acetate (pH 7.5), 10% glycerol, 0.05% Tween-20, and 0.5 mM DTT using 2.5 nM ssDNA (8, 12, 14, 16, 20, and 30 nt) titrated with hexameric concentrations of FL Twinkle and CTD. *A*, length dependence binding titration curves for FL Twinkle on a series of ssDNA lengths. *B*, length dependence binding titration curves for CTD on a series of ssDNAs. The equilibrium dissociation constant (*K*_*D*_) *versus* ssDNA length for FL Twinkle (*C*) and CTD (*D*). The primary structure schematic of each construct with included amino acid residues is displayed above each respective binding titration graph. Each curve fit to one-site binding hyperbolic equation and standard error of fit are shown. CTD, C-terminal domain; FL, full length.

Ring-shaped helicases generally bind ssDNA in the central channel, and the structure of the archetypal T7 gp4 helicase shows that each subunit contacts two nucleotides; thus, the minimal DNA-binding length is 12 nt per hexamer (13, 30). We made a series of ssDNA ligands of random sequence from 8-nt to 30-nt (Table S1) to determine the DNA-binding affinity and the minimal DNA length required to bind FL Twinkle and CTD. Each DNA contained a fluorescein moiety at the 3′-end, which enabled us to measure the *K*_*D*_ values using fluorescence anisotropy titrations, which unlike mobility shift assays are not disruptive and measures binding under equilibrium conditions. An increasing protein concentration was added to a constant amount of fluorescein-labeled ssDNA, and the resulting increase in anisotropy was plotted as binding curves that were fit to a 1:1 binding equation to obtain the *K*_*D*_ values (Fig. 3). The *K*_*D*_ values were measured in 50 mM Tris acetate buffer under two salt conditions, one without added NaCl and one with 50 mM NaCl (Table 1).

**Table 1:** Equilibrium dissociation constants (*K*_*D*_) for FL Twinkle, CTD, and NTD constructs binding to ssDNAs of increasing lengths determined from fluorescence anisotropy–based titrations fit to a one-site binding hyperbolic equation

DNA length	FL Twinkle	CTD	NTD
*K*_*D*_ (nM) no salt added			
8	9.0 ± 4.9	116 ± 41	—
12	5.2 ± 1.0	21.7 ± 3.0	141 ± 25
14	3.3 ± 0.7	8.8 ± 1.5	121 ± 26
16	2.8 ± 0.7	5.2 ± 1.4	105 ± 24
20	1.9 ± 0.5	2.2 ± 0.7	18.0 ± 7.0∗, 1200 ± 640
30	1.6 ± 0.3	1.7 ± 0.5	17.5 ± 4.0∗, 730 ± 450
*K*_*D*_ (nM) + 50 mM NaCl			
8	10 ± 12	—	—
12	53 ± 17	—	—
14	39 ± 16	128 ± 84	—
16	22.6 ± 5.1	117 ± 45	—
20	8.8 ± 1.3	50 ± 11	440 ± 260
30	2.6 ± 0.5	4.5 ± 0.9	264 ± 62

The ∗ represents the tight binding phase *K*_*D*_ value from fit to sum of two hyperbolas.

FL Twinkle and CTD bind DNA of various lengths with nanomolar *K*_*D*_ values, but overall, the affinity of CTD for the DNA is weaker than FL Twinkle, particularly DNAs from 8-nt to 16-nt in length (Table 1). Each protein shows a length dependency. The 8-nt DNA binds weakly to both FL Twinkle and CTD, but the affinities increase with increasing ssDNA length (Fig. 3, *A* and *B*). A plot of *K*_*D*_ *versus* DNA length indicates that the critical DNA-binding length is 14 to 16 nt for both proteins (Fig. 3, *C* and *D*). If each Twinkle subunit binds to 2-nt of DNA as with T7 gp4 helicase, then this result suggests that CTD and FL Twinkle assume a heptameric/octameric structure with the DNA. It would be overreaching to imply certainty in the oligomeric state from these experiments. However, the similar critical DNA-binding lengths of CTD and FL Twinkle suggest similar oligomeric states of the two constructs in the presence of DNA. Thus, the larger oligomers of CTD observed in the gel filtration experiments (Fig. 2, *B* and *C*) reorganize into FL Twinkle–sized oligomers in the presence of DNA.

### DNA binding to Twinkle CTD is more salt sensitive than FL Twinkle

**Figure 4:**
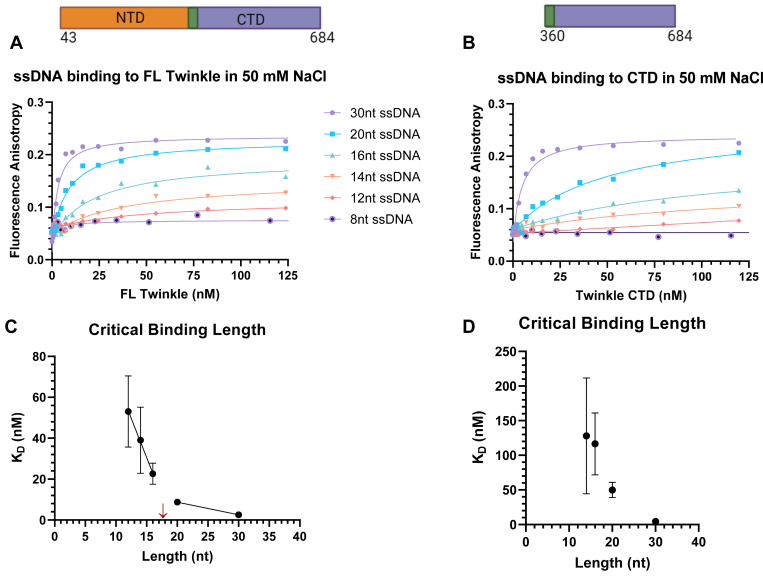
**ssDNA binding to FL Twinkle and CTD in 50 mM NaCl.** The fluorescence anisotropy–based titrations were carried out in same buffer as for Figure 3 with 50 mM NaCl added. Each curve was fit to one-site binding hyperbolic equation. *A*, length dependence binding titration curves for FL Twinkle for the series of ssDNA lengths. *B*, length dependence binding titration curves for CTD for the series of ssDNAs. The equilibrium dissociation constant (*K*_*D*_) *versus* ssDNA length for FL Twinkle (*C*) and CTD (*D*). The primary structure schematic of each construct with included amino acid residues is displayed above each respective binding titration graph. Each curve fit to one-site binding hyperbolic equation and standard error of fit are shown. CTD, C-terminal domain; FL, full length.

Adding 50 mM NaCl weakened the DNA-binding affinities of both CTD and FL Twinkle (Fig. 4). However, salt addition had a more pronounced effect on CTD than the FL Twinkle. For example, under no NaCl conditions, CTD binds to 12-nt with *K*_*D*_ of 22 nM, which is fourfold weaker than FL Twinkle *K*_*D*_ (∼5 nM). In 50 mM NaCl, CTD showed barely any binding to 12-nt DNA, whereas FL Twinkle bound with 50 nM *K*_*D*_. Salt addition also reduced the binding affinity of the longer 14 to 20 nt oligos by 14- to 22-fold for CTD as opposed to 5 to 10 for FL Twinkle. These DNA-binding studies demonstrate that CTD can bind DNA on its own, but NTD is necessary for stable binding. Thus, NTD directly or indirectly contributes to DNA binding in FL Twinkle.

### Twinkle NTD binds to ssDNA

**Figure 5:**
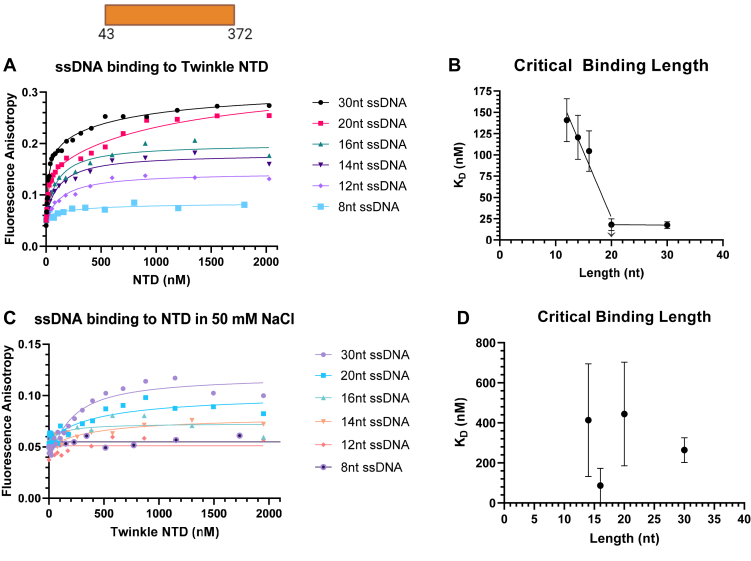
**Length-dependent binding of ssDNAs to Twinkle NTD.** The fluorescence anisotropy–based titrations were carried out as detailed for Figures 3 and 4. *A*, DNA-binding titration curves of NTD for a series of ssDNA in no salt added conditions. *B*, NTD *K*_*D*_*versus* ssDNA length in no salt added conditions. *C*, DNA-binding curves for NTD in 50 mM NaCl conditions. *D*, NTD *K*_*D*_*versus* ssDNA length in 50 mM NaCl conditions. Each curve fit to one-site binding hyperbolic equation except for 30 and 20 nt ssDNA in no salt added conditions, which were fit to two-site binding hyperbolic equation, and standard error of fit are shown. NTD, N-terminal domain.

To determine whether NTD contributes directly to DNA binding, we used fluorescence anisotropy–based titrations to measure the *K*_*D*_ values of NTD for the same series of 8-nt to 30-nt long ssDNAs we used to evaluate FL Twinkle and the CTD. A previous study using gel mobility shift assay concluded that NTD does not bind to DNA (25). Interestingly, in our hands, NTD has a DNA-binding activity that can be quantified by equilibrium titrations (Fig. 5). The NTD displayed negligible binding to the 8-nt DNA, but the 12-nt to 30-nt DNAs showed nanomolar *K*_*D*_ values with affinities increasing with increase in DNA length. Interestingly, the 20-nt and 30-nt DNAs showed a biphasic behavior in binding titrations (Fig. S3). The high-affinity binding mode displays *K*_*D*_ values around 20 nM and the low-affinity mode around 730 to 1200 nM (Table 1). The biphasic behavior suggests that two NTDs are involved in binding 20 to 30 nt DNA.

The DNA-binding activity of NTD was acutely salt sensitive. The 50 mM NaCl competed effectively with the binding of short DNAs from 8 to 16 nt, and the salt decreased the affinity of the 30-nt DNA by 15-fold, from ∼20 to ∼260 nM. These results demonstrate that NTD has a DNA-binding activity that can contribute directly to the overall high affinity and stability of the FL Twinkle on DNA.

### The Twinkle CTD hydrolyzes ATP and unwinds duplex DNA but less efficiently than FL Twinkle

**Figure 6:**
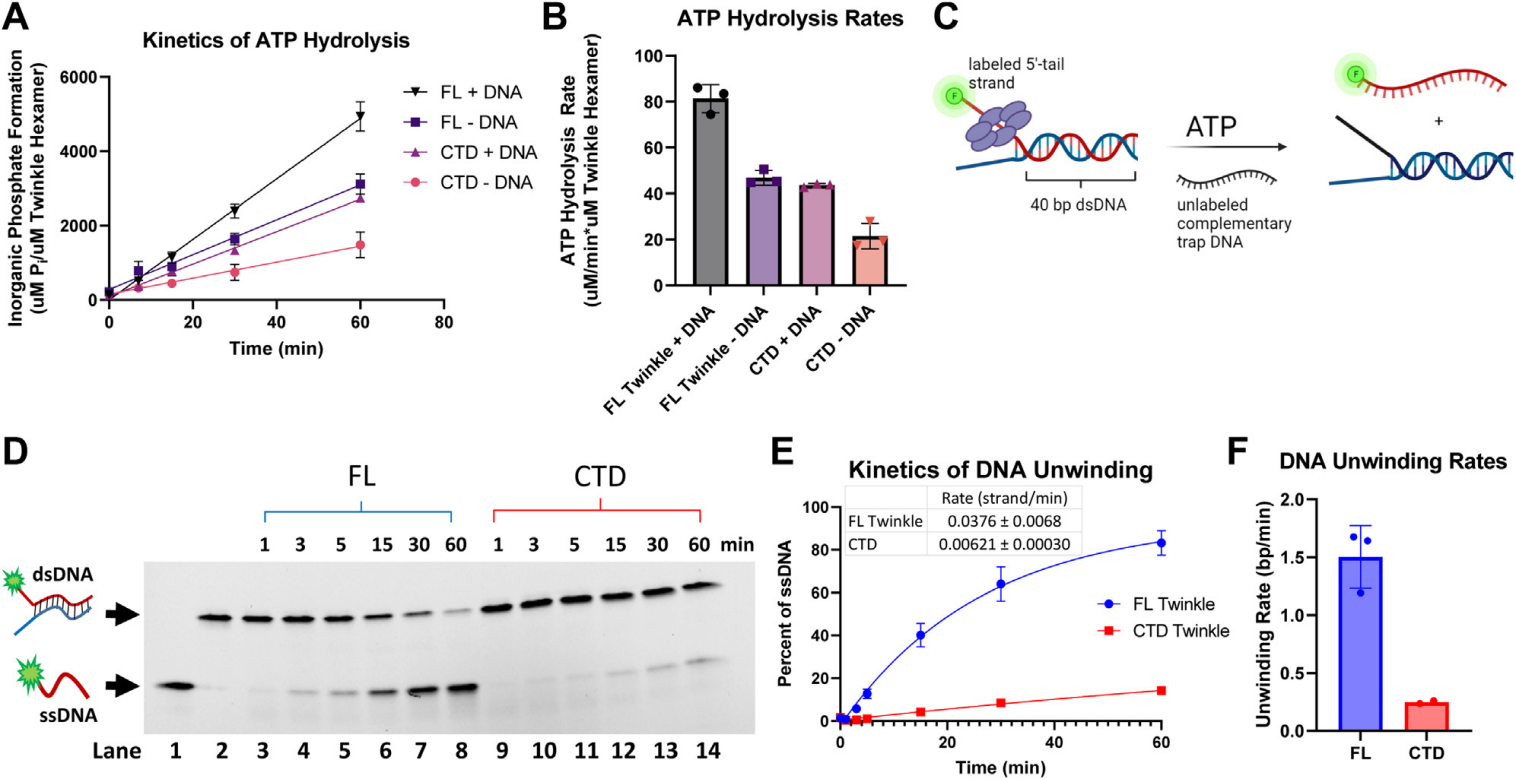
**ATP hydrolysis and DNA unwinding kinetics of CTD and FL Twinkle.***A*, time courses of ATP hydrolysis reaction for CTD and FL Twinkle with and without DNA. ATP hydrolysis was measured using 30 nM CTD or FL Twinkle (hexamer) with and without 2.5 nM M13 ssDNA molecules in 8 mM magnesium acetate and 1 mM ATP spiked with [γ-^32^P] ATP. *B*, the ATP hydrolysis rates from three replicates from *A* are shown with the standard deviations. *C*, schematics of the unwinding reaction showing 5′-ssDNA tail–bound Twinkle catalyzing the release of fluorescent 5′-tail DNA from a 40-bp duplex in the presence of ATP and trap DNA. The unwinding assay was carried out using the same buffer with 10 nM forked DNA, 4.5 mM ATP, 8 mM magnesium acetate, 55.5 nM Twinkle hexamer, and 280 nM trap DNA. *D*, representative image of a 4 to 20% native polyacrylamide gel showing the time course of ssDNA formation by FL Twinkle and CTD. *Lane* 1: free 5′-tail ssDNA, *lane* 2: free forked DNA, and *lanes* 3 to 8 and *lanes* 9 to 14 show time courses of forked DNA unwinding by FL Twinkle and CTD, respectively. *E*, DNA unwinding kinetics showing proportion of 5′-tail release for the FL Twinkle and CTD fit to a one-phase exponential equation, rate displayed. *F*, the rate of DNA unwinding in base pairs/minute was calculated by multiplying the rate of strands unwound per minute by the number of base pairs in the fork, graphed with individual replicates as points and error bars representing SD. CTD, C-terminal domain; FL, full length.

The ATPase motifs are present in the CTD, and these motifs form an active site at the subunit interface in the ring structure (10). The ATPase activity is essential for supporting translocation and unwinding activities of the helicase. The time course of ATP hydrolysis shows that both FL Twinkle and CTD hydrolyze ATP in the absence and presence of DNA (Fig. 6*A*). Compared with FL Twinkle, the ATP hydrolysis rate of CTD was only twofold lower (Fig. 6*B*). Thus, NTD loss lowers the ATPase activity of the CTD, but the impairment is moderate. Adding M13 ssDNA increases the ATPase rate of both FL Twinkle and CTD by twofold. These results indicate that the isolated CTD can assemble into ATPase-active oligomers both in the presence and absence of DNA.

Twinkle is a 5′-3′ helicase and assembles on the 5′-ssDNA tail to initiate DNA unwinding of the duplex region (7, 11, 31). We tested the DNA unwinding activity of FL Twinkle and CTD using a 40-bp forked DNA (Table S2) under single-turnover conditions (Fig. 6*C*). The 40-bp fork DNA contained a 28-nt 3′-tail and a 35-nt 5′-tail labeled with fluorescein to monitor DNA strand separation using a native gel assay. The helicase reactions were carried out by incubating Twinkle with the forked DNA and adding ATP, Mg^2+^, and an excess of trap DNA to capture the unwound strand (unlabeled lower strand) and the free/dissociated Twinkle. FL Twinkle unwinds the fork DNA at a rate of 1.5 bp/min, but the CTD shows a weak DNA unwinding activity with a rate of 0.25 bp/min (Fig. 6, *D*–*F*). This indicates that deletion of NTD has a more drastic effect on the helicase activity than the ATPase activity. Thus, the isolated CTD can assemble into ATPase active oligomers in the absence of NTD, but NTD is necessary to functionally couple the ATPase activity to DNA unwinding.

### Twinkle CTD supports strand-displacement DNA synthesis by Polγ on a short replication fork DNA

**Figure 7:**
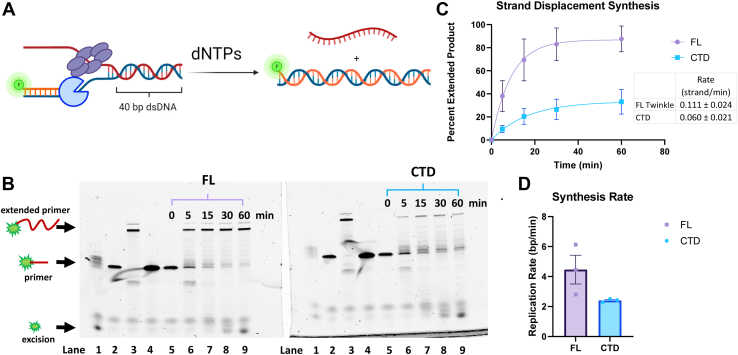
**Strand displacement DNA synthesis activity of Polγ with and without FL Twinkle or CTD.***A*, schematic shows the strand displacement DNA synthesis assay with fluorescein-labeled primer to monitor primer extension by the denaturing gel assay. Reactions were carried out with 300 nM Twinkle hexamer or CTD hexamer, 100 nM forked DNA, 200 nM Polγ, 300 μM dCTP, 100 μM remaining dNTPs, and 4 mM ATP. *B*, representative image of the 15% TBE–urea gel resolving the starting primer and extended primer from DNA synthesis. Samples from reactions with FL Twinkle or CTD were loaded in identical lanes in the two gels. *Lane* 1: fork DNA + Polγ reaction, *lane* 2: forked DNA alone, *lane* 3: Polγ + primer-template reaction, *lane* 4: free primer, and *lanes* 5 to 9: reactions with Polγ and FL Twinkle or CTD. *C*, proportion of the extended primer with Polγ and FL Twinkle or CTD (error bars represent three replicates). The kinetics fit to a single exponential equation to provide a composite rate of DNA synthesis over the 40-bp duplex (*solid lines*). *D*, rates of DNA synthesis in base pairs/minute, calculated by multiplying the rate of DNA strand synthesis per minute by the number of base pairs in the fork, for FL Twinkle and CTD with individual replicates as points and error bars representing standard deviation. CTD, C-terminal domain; FL, full length; Polγ, DNA polymerase γ; TBE, Tris–borate–EDTA.

Twinkle, Polγ, and mtSSB have been shown to catalyze rolling circle leading strand DNA synthesis (8). Herein, we used a short 40-bp replication fork consisting of a 30-nt primer to measure wildtype Polγ′s strand-displacement DNA synthesis activity with and without Twinkle (Table S2 and Fig. 7*A*). We used wildtype Polγ, which has proofreading 3′-5′ exonuclease activity, because the exonuclease minus Polγ has been shown to have intrinsic strand-displacement synthesis activity in the absence of Twinkle (32, 33). The 45-nt 5′-tail of the forked DNA is long enough to bind one Twinkle oligomer; hence, mtSSB was not added to the reactions. In the absence of Twinkle, Polγ fills the 4-nt ssDNA gap between the 3′-end of the primer and the start of the 40-bp duplex region in the replication fork but does not carry out strand-displacement DNA synthesis (Fig. 7*B*, *lane* 1). When FL Twinkle was added, Polγ could strand displace and fully extend the primer to the FL product (Fig. 7*B*, *lanes* 6–9), demonstrating that Twinkle and Polγ can catalyze DNA unwinding synthesis without mtSSB on a short fork DNA. We also observed an accompanying excision reaction, which degraded part of the primer at the start. Interestingly, despite its previously characterized poor unwinding activity (Fig. 6, *D*–*F*), the CTD could catalyze unwinding synthesis with Polγ on the short replication fork DNA. The primer extension yield in the CTD reactions was threefold lower compared with FL Twinkle (Fig. 7*C*), and the DNA synthesis rate of Polγ and CTD was twofold lower (Fig. 7*D*). These results indicate that Polγ can functionally couple with CTD to catalyze strand-displacement synthesis, albeit with lesser efficiency.

### Twinkle NTD is necessary for supporting rolling circle DNA synthesis by Polγ

**Figure 8:**
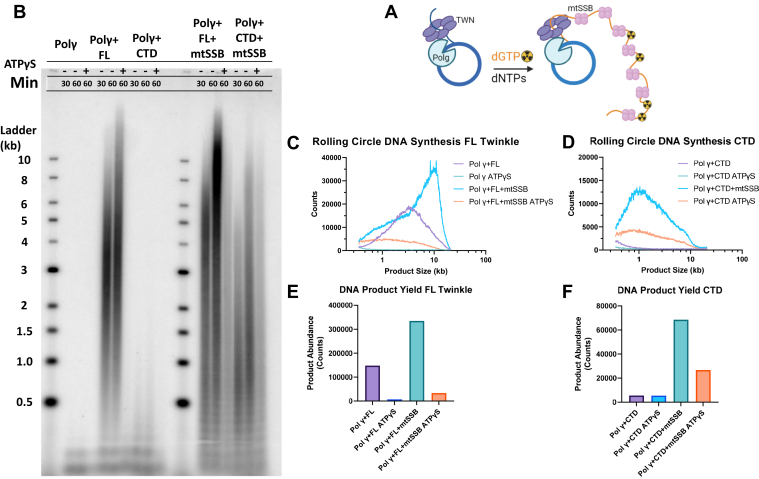
**Rolling circle DNA synthesis on the 70-bp minicircle fork DNA.***A*, schematic shows *rolling circle* DNA synthesis on the 70-bp minicircle forked DNA with Twinkle, Polγ, and mtSSB. Reactions were carried out using 20 nM FL Twinkle or CTD hexamer, 20 nM wildtype Polγ, 250 nM mtSSB when added, 10 nM 70 bp minicircle forked DNA, 2 mM ATP, 250 μM dNTPs, 25 μM dGTP spiked with [α-^32^P]dGTP at 37 °C. *B*, representative image of an 0.8% alkaline agarose gel showing the DNA products from the rolling circle DNA synthesis with the DNA size marker ladder. *C*, quantified counts of the 60 min reaction as a function of product length with FL Twinkle and Polγ with and without mtSSB and ATPγS. Product lengths were determined from the calibration curve generated from the DNA ladder run in the same gel. *D*, quantified counts of the 60 min reaction as a function of product length with CTD and Polγ with and without mtSSB and ATPγS. *E*, total DNA product counts for each reaction containing FL Twinkle and Polγ with and without mtSSB and ATPγS. *F*, the total product counts for each reaction containing CTD and Polγ with and without mtSSB and ATPγS. CTD, C-terminal domain; FL, full length; mtSSB, mitochondrial single-stranded DNA-binding protein; Polγ, DNA polymerase γ.

The entire mitochondrial genome of ∼16 kb is copied by the leading strand mt replisome complex consisting of Twinkle, Polγ, and mtSSB (34). Such long stretch DNA synthesis can be assessed *in vitro* using rolling circle DNA synthesis on a 70-bp minicircle DNA (8), which we used to assess the replication activity of Polγ with CTD (Fig. 8*A*). The radioactively labeled reaction products were resolved on an alkaline agarose gel to assess the size and yields of the synthesized DNA (Fig. 8*B* and Table S3). As shown by the markers, the alkaline agarose gel separates DNA products from a few hundred bases in length to >10 kilobases. Polγ alone does not catalyze rolling circle synthesis on its own, and no detectable DNA products were observed in those reactions. However, in the presence of FL Twinkle, Polγ was able to make >10 kb-sized products with an average size around 4 kb (Fig. 8*C*). This result demonstrates that FL Twinkle and Polγ can cooperatively catalyze processive strand-displacement DNA synthesis even in the absence of mtSSB. Substituting FL Twinkle with CTD reduced DNA synthesis drastically (Fig. 8*D*); faint bands of 0.5 to 1 kb length products were detected but in meager yields (Fig. 8, *D* and *F*). These results indicate that CTD requires the activity of the NTD to catalyze strand-displacement DNA synthesis over long DNA stretches. Addition of the NTD protein to the CTD and Polγ reaction did not improve rolling circle DNA synthesis as compared with CTD alone, indicating that the NTD does not effectuate its function in *trans* (Fig. S4).

The leading strand DNA synthesis reaction by the mt replisome produces long stretches of ssDNA covered with mtSSB *in vivo* (35). The *in vitro* rolling circle DNA synthesis also produces long stretches of ssDNA (1 kb to >10 kb) that must be covered with mtSSB to prevent secondary structure formation and off target Twinkle or Polγ binding. Thus, addition of mtSSB increased the average sizes of DNA synthesized by FL Twinkle and Polγ from 4 kb to 11 kb and increased the yield of DNA synthesis by twofold (Fig. 8, *C* and *E* and Table S3). Remarkably, adding mtSSB stimulated the CTD and Polγ reactions by a significant 12-fold and increased the observed size of synthesized DNA from a few hundred bases to ∼10 kb lengths with an average size of ∼2 kb (Fig. 8, *D* and *F* and Table S3). This dramatic increase in DNA synthesis processivity with CTD and mtSSB was unexpected. To tease out the contribution of CTD *versus* mtSSB to DNA unwinding, we substituted ATP with ATPγS, which should inhibit the contribution of the motor activity from the CTD but not the energetically passive activity of the mtSSB (Fig. 8, *B* and *D*). These experiments show that about one-third of synthesis products are from Polγ and mtSSB catalysis, and the remaining two-thirds are from CTD, Polγ, and mtSSB catalysis (Fig. 8*F* and Table S3). Thus, mtSSB stimulates the unwinding activity of CTD and partially compensates for the loss of NTD by binding to the nascent DNA behind the CTD.

## Discussion

In this study, we have determined the role of the NTD of Twinkle by biochemically characterizing the isolated NTD and CTD domains and comparing their activities to FL Twinkle. Human Twinkle is homologous to bacteriophage T7 gp4 helicase–primase, whose NTD harbors a primase function necessary for Okazaki fragment synthesis (18, 19, 20, 21). The prevailing model of human mtDNA replication is the asynchronous strand-displacement mechanism, where the heavy strand of the mtDNA is copied continuously from RNA primers made by the mitochondrial RNA polymerase POLRMT without Okazaki fragment synthesis (24, 34, 35, 36, 37), although alternative models exist (38). Because lagging strand synthesis does not occur concomitant with leading strand synthesis, frequent priming is unnecessary, which could be the reason for the evolutionary loss of primase function in human Twinkle. This raises the question of why the NTD, constituting nearly half of the protein by mass and volume, is maintained in human Twinkle. A previous study demonstrated that NTD is necessary, and its partial deletion impairs rolling circle DNA synthesis with Polγ and mtSSB (25). However, since no definitive activity was shown for the NTD, its precise role was unclear.

In this study, we demonstrate that Twinkle NTD has a DNA-binding activity, and it also lends stability and uniformity to Twinkle oligomers. We show that while the CTD and linker region construct can oligomerize, it elutes as a broad peak with higher order species from gel filtration compared with FL Twinkle, which elutes as a species consistent with the expected size of previously observed Twinkle, a hexamer or heptamer (9, 29). The recent structure of a disease-causing Twinkle mutant in heptameric/octameric form (10) and the AlphaFold subunit structure of Twinkle show that NTD makes many interactions with the linker region (Fig. S1), which supports the role of NTD in bolstering the ring structure. The linker and NTD interface are also where many disease-causing mutations are found (Fig. S1). Many of these mutants perturb assembly and generate larger oligomeric rings or broken rings (10, 29, 39).

The defect in DNA binding because of NTD deletion has a more significant effect on the helicase function of CTD rather than its ATPase activity. Because the ATPase site lies at the subunit interface, the formation of oligomer would be sufficient for ATP hydrolysis. On the other hand, the helicase function requires coordination between DNA binding–release steps and ATP binding–hydrolysis steps in each subunit (40, 41). Hence, any defect in DNA-binding activity can decouple the ATPase activity from the motor function resulting in poor helicase function. For example, T7 gp4 binds DNA more weakly in the presence of ATP compared with dTTP (42); and although, ATP is hydrolyzed by T7 gp4, it does not support DNA unwinding, (43, 44). Processive unwinding with little slippage was observed with dTTP in single-molecule unwinding experiments with T7 gp4, whereas repeated unwinding and slippage events were observed with ATP (42). Thus, in Twinkle, the loss in DNA-binding energy because of lack of NTD could increase helicase slippage events, explaining the more substantial impairment in unwinding function compared with the ATPase activity. Interestingly, CTD and Polγ together were able to catalyze DNA unwinding synthesis reaction on the short fork with product yields higher than the unwinding reaction with CTD alone. Thus, concomitant DNA synthesis by Polγ can partially restore the unwinding defect caused by NTD deletion. Such cooperativity has been observed in T7 replisome, where the DNA polymerase accelerates the unwinding activity of T7 gp4 (40, 41, 45).

**Figure 9:**
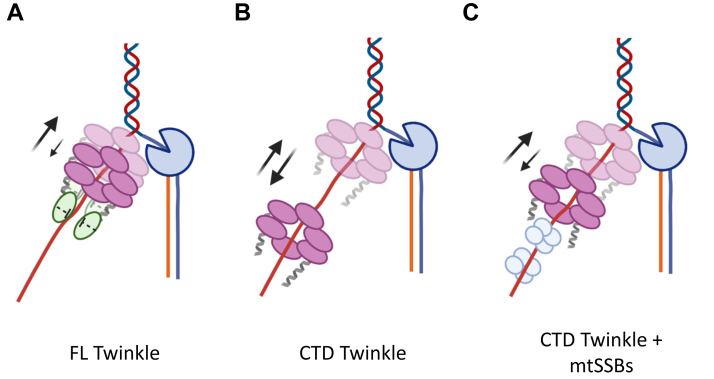
**Proposed doorstop mechanism for the role of NTD in supporting processive translocation for DNA unwinding as part of a replisome with Polγ and mtSSBs.***A*, the NTD binding to the DNA emerging from the central channel of the CTD domains in FL Twinkle minimize backward motion (*down arrow*) promoting forward motion (*up arrow*). *B*, the CTD at the replication fork has more significant backward motion. *C*, the mtSSB molecules binding the DNA behind the CTD ring prevent excessive backward movements. CTD, C-terminal domain; FL, full length; mtSSB, mitochondrial single-stranded DNA-binding protein; NTD, N-terminal domain; Polγ, DNA polymerase γ.

Although CTD and Polγ could catalyze strand-displacement synthesis on the short replication fork, the two could not catalyze kilobase length DNA products in rolling circle DNA synthesis as with FL Twinkle. This demonstrates the critical need for the NTD in processive unwinding to support genome-sized DNA products in leading strand synthesis. We were intrigued when mtSSB was able to partially restore the synthesis of long DNA products by Polγ and CTD. The mtSSB also stimulated strand-displacement synthesis activity of the FL Twinkle but more significantly activated the CTD reactions. The tetrameric mtSSB binds ∼30 nt ssDNA with a high affinity (46) and is expected to coat the nascent ssDNA as it emerges from the central channel of the Twinkle molecule unwinding DNA at the fork junction (35). The CTD subunits are undergoing significant conformational changes unwinding the base pairs at the fork junction and moving the DNA in the central channel all while possessing reduced ring stability and reduced DNA affinity. The mtSSB binding behind Twinkle could minimize the backward slippages of the CTD and prevent DNA from dissociating (Fig. 9). We propose that the DNA-binding activity of the NTD has a similar role in activating and increasing the processivity of DNA unwinding by Twinkle. In this way, the NTD serves as a “doorstop” and holds the DNA emerging from the CTD near the central channel preventing the DNA from dissociating and CTD from slipping backward. When linked to CTD as a part of the FL Twinkle, two or three NTDs can cooperate and bind DNA strongly, albeit nonspecifically to ensure NTDs do not inhibit DNA translocation. As the addition of NTD in *trans* failed to complement CTD in supporting rolling circle DNA synthesis by Polγ (Fig. S4), we speculate that the assistance of the NTD requires the covalent linkage to CTD, which allows the NTDs to be positioned directly adjacent to the extruded strand for coordinated binding and prevention of the backward motion of Twinkle. Thus, our studies reveal that even though the NTD of the human Twinkle has lost its primase function, it has retained its DNA-binding activity to enhance the helicase functions of the CTD.

## Experimental procedures

### Nucleic acid substrates

Oligodeoxynucleotides were custom-synthesized with either 5′- or 3′-end fluorescein inverted T and biotin modifications and purified by HPLC (Integrated DNA Technologies). The 70-nt minicircle DNA was prepared by ligation reaction and assembled into a fork DNA, as described previously (47). DNA concentration was determined from the absorbance at 260 nm and the corresponding molar extinction coefficients.

### Protein purification

#### PolγA

The catalytic and accessory subunits of human mtDNA polymerase gamma (PolγA and PolgγB, respectively) were purified as reported previously (48). Histidine-tagged PolγA subunit lacking 1 to 29 putative mitochondrial localization signal sequence and 10 of the 13 sequential glutamine residues (amino acid residues 43–52) was expressed in Sf9 insect cells. Briefly, Sf9 cells expressing PolγA were lysed by gently stirring the cell pellets in lysis buffer consisting of 70% of lysis buffer P (0.32 M sucrose, 10 mM Hepes, pH 7.5, 0.5% [v/v] NP-40, 3 mM CaCl_2_, 2 mM magnesium acetate, EDTA-free protease inhibitor tablet [Roche], and 5 mM DTT). The centrifuged cell lysate was purified using nickel (Ni) affinity chromatography (HisTrap HP; Cytiva), followed by purification on heparin column (HiTrap heparin HP affinity column). The resulting protein was further purified to homogeneity by passing it through Superose 6 gel filtration column (Cytiva).

#### PolγB

Histidine-tagged PolγB was expressed in *Escherichia coli* Rosetta (DE3) cells. Cell pellets were lysed in 20 mM Hepes (pH 8.0), 300 mM KCl, 5% glycerol, 0.1% Triton X-100, one tablet of EDTA-free protease inhibitor per 50 ml buffer, 0.1 mg/ml lysozyme, and 10 mM beta-mercaptoethanol using sonication, treated with 0.1% final concentration of polyethyleneimine (PEI), and supernatant was applied to a prepacked Ni affinity column (HisTrap HP) pre-equilibrated with Ni buffer (20 mM Hepes [pH 8.0], 300 mM KCl, and 5% glycerol). The bound protein was eluted with a continuous 20 to 500 mM imidazole gradient. The eluted fractions enriched in PolγB were pooled, diluted to adjust KCl concentration to 60 mM, and loaded on a cation exchange chromatography column (HiTrap SP HP Column) pre-equilibrated with SP column low salt buffer containing 20 mM Hepes (pH 7.5), 60 mM KCl, 5% glycerol, and 5 mM beta-mercaptoethanol. The column was washed with the low KCl buffer to remove loosely bound contaminants. The bound PolγB was then eluted with a continuous gradient of 60 mM to 1 M KCl. The collected fractions with eluted PolγB were analyzed using SDS-PAGE, and the fractions with purified protein were pooled. The purified protein was diluted with SP column low salt buffer to achieve final KCl concentration of 100 mM KCl. Purified proteins were concentrated using Amicon Ultra Centrifugal Filters with molecular weight cutoff 10,000 (MilliporeSigma). Concentrated proteins were stored at −80 °C. To make PolγAB complex, PolγA and PolγB were mixed in molar ratio of 1:2.

#### Human mtSSB

mtSSB cloned in pET11a plasmid was expressed in *E. coli* BL21 (DE3) as reported previously (49). Expressed mtSSB was purified by closely following the protocol described by Longley *et al.* (50). Briefly, mtSSB expressing bacterial cells were lysed using sonication, and cell lysate was centrifuged to obtain supernatant with soluble recombinant protein. mtSSB was purified to homogeneity on blue sepharose (HiTrap blue HP), cation exchange (HiTrap SP HP), and anion exchange (HiTrap Q HP) chromatographic columns (all from Cytiva).

#### Twinkle CTD

SUMO-CTD (amino acids 360–684) cloned in pET28 SUMO expression vector was expressed in *E. coli* Rosetta 2 DE3 pLacI cells (50 mg/l kanamycin). A single colony was grown at 37 °C in 150 ml of LB containing 50 mg/l kanamycin for 15 h and inoculated into 1 l LB containing 50 mg/l kanamycin, grown at 37 °C to an absorbance of 0.7, and induced with IPTG (0.2 mM) for ∼15 h at 16 °C. The cells were suspended in lysis buffer (50 mM NaH_2_PO_4_ [pH 8.0], 1 M NaCl, 1 mM EDTA, 1 mM Tris(2-carboxyethyl)phosphine [TCEP], 0.1% Triton X-100, 0.5% NP-40, 0.2 mg/ml lysozyme, and Roche protease inhibitor tablets) and sonicated three times (amplitude 30, 3 min cycle with 10 s on, 10 s off bursts) on ice, centrifuged (15,500*g* for 1 h) to obtain the supernatant, which was treated with PEI to a final concentration of 0.5% to remove nucleic acids in the pellet after centrifugation. The proteins in the supernatant were precipitated by ammonium sulfate at 65% saturation and resuspended in buffer A (50 mM NaH_2_PO_4_ [pH 8.0], 10% glycerol, 0.4 M NaCl, 10 mM imidazole, 0.1% Triton X-100, 0.5% NP-40, and 5 mM DTT). The solution was incubated with Qiagen nickel–nitrilotriacetic acid agarose bead resin for 1 h, beads were washed two times with buffer A, and spun at 1000*g* for 1 min at 4 °C. The supernatant was removed, and protein was eluted with buffer A containing 200 mM NaCl and 500 mM imidazole. The eluted protein was mixed with ULP1 SUMO-protease and dialyzed for 12 h in buffer A with 200 mM NaCl and applied to a prepacked 1 ml Ni sepharose high performance (HP) affinity resin column in the same buffer. The flow-through was applied to a prepacked 1 ml HiTrap heparin HP column in buffer 50 mM Tris–Cl (pH 7.9), 10% glycerol, 200 mM NaCl, 1 mM TCEP, and eluted with a 30 ml gradient of 200 to 1500 mM NaCl. Fractions with pure CTD protein were pooled and concentrated using an Amicon Ultra-2 Centrifugal Filter unit with molecular weight cutoff 10,000 (MilliporeSigma) to ∼39 μM of monomeric Twinkle and measured for 260/280 ratio using a Nanodrop (Fig. S2*B*, *lane* 2 and Fig. S2*D*).

#### Twinkle NTD

SUMO-NTD (amino acids 43–372) was cloned into pET 28 SUMO expression vector and expressed in BL21 DE3 RIL *E. coli* cells using methods described previously for the CTD. The lysed cell supernatant was applied to a prepacked 5 ml Ni sepharose HP affinity resin column in buffer B (25 mM Tris–Cl [pH 8.0], 10% glycerol, and 10 mM imidazole) containing 400 mM NaCl and eluted using a gradient 250 ml gradient from 0 to 400 mM imidazole. The NTD containing fractions were combined and mixed with ULP1 SUMO-protease and dialyzed for 12 h in buffer B with 200 mM NaCl. The dialyzed solution was applied to a prepacked 1 ml Ni sepharose HP affinity resin column in buffer B with 200 mM NaCl, and the flow-through containing cleaved NTD was slowly diluted while stirring at 4 °C with buffer containing 20 mM NaH_2_PO_4_ (pH 7.7), 10 mM NaCl, 0.5 mM EDTA, 10% glycerol, and 1 mM DTT (buffer C) to bring the NaCl concentration to ∼100 mM NaCl. The clarified solution applied to a prepacked 1 ml HiTrap heparin HP column in buffer C with 100 mM NaCl and eluted with 200 ml gradient from 100 to 415 mM NaCl. The pure NTD fractions were combined and measured to be ∼26 μM (monomer NTD concentration) (Fig. S2*B*, *lane* 3 and Fig. S2*E*).

#### FL Twinkle

FL Twinkle (amino acids 43–684) construct was previously prepared in our laboratory (12). FL Twinkle was expressed as a SUMO fusion in *E. coli* Rosetta 2 DE3 pLacI cells, which were grown as described for the CTD, and lysed in buffer D (50 mM Tris–Cl [pH 7.1], 600 mM KCl, 1 mM EDTA, 10% glycerol, 0.1% Tween, and 0.2 mM TCEP) containing Roche protease inhibitor tablets using a cell homogenizer. The clarified lysate was treated with PEI and ammonium sulfate as described for CTD, and the ammonium sulfate pellet was dialyzed against buffer D for 12 h before applying to a prepacked 5 ml Ni sepharose HP affinity resin column. Twinkle was eluted using a 40 ml gradient of imidazole from 0 to 500 mM in buffer D. The fractions containing SUMO-Twinkle were combined, mixed with ULP1 SUMO-protease, and dialyzed for 12 h in buffer D with 300 mM KCl, 5 mM EDTA, and 5 mM DTT instead of the TCEP and for another 2 h with additional fresh buffer. The dialyzed fractions were applied to a prepacked 1 ml Q sepharose fast flow column in buffer D containing 150 mM KCl, 5 mM EDTA, and 1 mM TCEP and eluted using a 20 ml gradient from 250 to 1.25 mM KCl. The fractions containing FL Twinkle were applied to a prepacked 1 ml HiTrap heparin HP column in buffer D with 150 mM NaCl, 5 mM EDTA, and 1 mM TCEP and eluted with an NaCl gradient from 150 mM to 1 M. The fractions containing FL Twinkle were measured for 260/280 ratio and those below 0.8 were combined and concentrated using an Amicon Ultra-2 Centrifugal Filter unit with molecular weight cutoff 10,000 to ∼39 μM (monomeric Twinkle concentration) (Fig. S2*B*, *lane* 1 and Fig. S2*C*).

### Evaluating oligomeric state by gel filtration chromatography

Size-exclusion chromatography was carried out using Superdex 200 Increase 10/300 GL Cytiva column at 4 °C. Bio-Rad protein standards were applied at 0.3 ml/min in running buffer as described to the chromatography column. The peak elution volumes (280 nm detection) were plotted against the logarithm of protein molecular weight to obtain the calibration curve. Oligomerization of FL Twinkle (15 μl of 38.5 μM monomeric concentration) was tested in buffer E (50 mM Tris–Cl [pH 7.1] and 5% glycerol) with 300 mM NaCl and 1 mM TCEP. Oligomerization of CTD (50 μl of 10 μM monomeric concentration) was tested in buffer E containing 600 mM NaCl. Oligomerization of NTD (500 μl, 61 μM monomeric concentration NTD) was tested in buffer C with 200 mM NaCl and 5% glycerol. Expected elution volume for a hexamer and a monomer of each construct was calculated using the calibration curve. Peak elution volume is displayed as a function of protein molecular weight using GraphPad Prism 9.0 (GraphPad Software).

### DNA binding using fluorescence anisotropy titrations

Serial diluted protein solution was mixed with fluorescein-labeled DNA (IDT; HPLC purified) at 2.5 nM concentration in buffer containing 50 mM Tris acetate (pH 7.5), 10% glycerol, 0.05% Tween-20, and 0.5 mM DTT with and without 50 mM NaCl. Fluorescence intensities were measured on the TECAN Spark plate reader at excitation wavelength 485 (20 nm bandwidth) and emission wavelength 535 (20 nm bandwidth). The anisotropy (*r*) was calculated from the parallel and perpendicular polarized light emission intensity (*I*) utilizing the equation: $r=\frac{I\|-I⊥}{\left(I\|+2*I⊥\right)}$ and plotted against protein concentration (*P*) (monomer for NTD, hexamer for CTD and FL Twinkle). The curves were fit to equation as follows: $Y=\frac{r_{\max}*\left[P\right]}{\left(K_{D}+\left[P\right]\right)}+Y_{0}$ for each binding titration curve with the exception of the NTD binding to 30 nt and 20 nt length ssDNAs in the absence of salt, which were fit to the equation as follows: $Y=\frac{r_{\max\;Hi}*\left[P\right]}{\left(K_{D\;1st}+\left[P\right]\right)}+\frac{r_{\max\;Low}*\left[P\right]}{\left(K_{D\;2nd}+\left[P\right]\right)}+Y_{0}$. Here, *Y*_*0*_ is the anisotropy of free DNA, and *rmax* is the anisotropy of protein-bound DNA. Protein binding curves are graphed using GraphPad Prism 9.0.

### Radiometric assay to measure ATP hydrolysis

The ATPase reactions were carried out in buffer F (50 mM Tris acetate [pH 7.5], 0.01% Tween-20, 1 mM EDTA, and 5 mM DTT) with and without 2.5 nM M13 ssDNA using 30 nM Twinkle hexamer, 8 mM magnesium acetate, and 1 mM ATP spiked with [γ^32^P]ATP. Reactions were quenched with 8 M formic acid, spotted on a PEI cellulose TLC, and developed in 0.4 M potassium phosphate (pH 3.4). The counts from the resolved ATP and inorganic phosphate spots were used to obtain the proportion of ATP hydrolysis with time. ATP hydrolysis and rates are graphed using GraphPad Prism 9.0.

### DNA unwinding assay

DNA unwinding assays were carried out in buffer F using 10 nM preannealed fluorescein-labeled DNA fork, 55.5 nM FL Twinkle or CTD hexamer, 4.5 mM ATP, and 8 mM magnesium acetate. Buffer components, ATP, DNA fork, and Twinkle are combined in solution A, and magnesium acetate and trap DNA (unlabeled upper strand, 280 nM final concentration) were combined in solution B. Solution A and B were mixed in equal volumes to begin the reaction at 30 °C. Portions of the reaction were removed at various time intervals, diluted 1:1 with quenching solution containing 100 mM EDTA (pH 8.0) and 1% SDS to stop active enzymatic activity, and denature the protein. The quenched samples were mixed 1:10 with loading buffer (15% Ficoll 400 in 0.3× Tris–borate–EDTA) loaded into a 4 to 20% TGX gradient gel running at a low voltage of 150 V while loading samples. The gel was run for approximately 2 h at 4 °C until the bromophenol blue dye in an otherwise empty lane reached near the bottom of the gel but did not run off. The gels were scanned for fluorescence on a GE Typhoon FLA 9000 Gel Scanner (GE Healthcare Bio-Scienses AB) at 600 V in the FAM mode and processed using ImageQuant TL 8.2 (GE Healthcare Bio-Scienses AB) in 1D gel analysis mode. The rolling ball method of background subtraction was used on the least sensitive (200) setting, background subtracted counts were graphed and analyzed in GraphPad Prism 9.0. The release of the fluorescently labeled strand was graphed as a proportion of ssDNA to total fluorescent DNA over time and fit to exponential equation, $Y=Y_{0}+A*\left(1-e^{\left(-k*x\right)}\right)$. The rate of ssDNA formation (k) in min^−1^ was determined and multiplied by the number of base pairs in the fork (40 bp) to obtain rate of unwinding in base pairs/minute.

### Strand-displacement DNA synthesis on short replication fork

Reactions contained 100 nM assembled primed fork DNA, 200 nM wildtype Polγ (A and B in 1:2 ratio), 300 nM FL Twinkle or CTD hexamer, 10 mM MgCl_2_, 4 mM ATP, 100 μM dNTPs, and 300 μM dCTP in buffer containing 50 mM Tris–Cl (pH 7.5), 40 mM NaCl, 10% glycerol, and 2 mM DTT. Buffer components, Twinkle, Polγ, fork DNA, and dCTP were combined into solution A over a series of additions and incubations. Polγ was added to primed fork DNA with dCTP (next nucleotide) and incubated on ice for 5 min in buffer without MgCl_2_. Twinkle or CTD was added, incubated on ice for 20 min, and then at 37 °C for 5 min before adding dNTPs, MgCl_2_, and ATP. Portions of reaction were removed and quenched at 5, 15, 30, and 60 min with 500 mM EDTA and 2 μM trap DNA (unlabeled upper strand). Samples were diluted 1:1 with 100% formamide, boiled for 5 min before being quickly transferred to ice, and loaded into a 15% Tris–borate–EDTA–urea denaturing gel and run at 250 V until bromophenol blue loaded into an otherwise empty lane migrated two-thirds of the way through the gel. Polγ incubated with the primer annealed only to the complementary lower strand without the upper strand present was used as a positive control for full extension, whereas the primer alone was used for a no extension control. The gels were scanned and analyzed as aforementioned, except they were fit to a one-phase exponential equation, $Y=Y_{\max}*\;\left(1-e^{\left(-k*x\right)}\right)$ using GraphPad Prism 9.0, the units were similarly displayed as min^−1,^ and converted to base pairs/minute as described previously.

### Rolling circle DNA synthesis assay

Reaction contained 20 nM Polγ, 10 nM 70 bp minicircle fork, 250 nM mtSSB tetramer, 20 nM FL Twinkle or CTD hexamer, 10 mM MgCl_2_, 2 mM ATP, 0.25 mM dATP, 0.25 mM dCTP, 0.25 mM dTTP, and 25 μM dGTP spiked with [α-^32^P]dGTP in 50 mM Tris-Cl (pH 7.5), 10% glycerol, and 2 mM DTT. Buffer components, minicircle fork, Polγ, and mtSSB were combined, in that order in solution A and allowed to incubate for 20 min at 37 °C. MgCl_2_, ATP, dNTPs, and [α-^32^P]dGTP were combined in solution B and added to solution A to begin the reaction. Portions of the reaction were removed at various time intervals, diluted 1:1 with quenching solution containing 100 mM EDTA (pH 8.0) to stop active enzymatic activity. Proteinase K was added to a final concentration of 0.1 mg/ml, and the samples were incubated for 45 min at 42 °C to digest any bound protein off of the DNA. The digested samples were mixed 1:10 with loading buffer (300 mM NaOH, 6 mM EDTA, 18% [w/v] Ficoll 400, 0.15% [w/v] bromophenol blue, and 0.25% xylene cyanol). The samples were loaded on 0.8% alkaline agarose gel with 1 kb NEB ladder labeled with [γ-^32^P] ATP using T4 polynucleotide kinase, and electrophoresed in running buffer containing 50 mM NaOH and 6 mM EDTA at 74 V and 4 °C for 15.5 h. The gel was fixed in 7% trichloroacetic acid, dried, and exposed to a phosphorscreen, scanned on a Typhoon scanner. Scans of the phosphor screen were analyzed with the “Analysis Toolbox” application in ImageQuant TL 8.2. A quantitative object line was drawn through the center of each lane longitudinally through the gel, and the counts along the length of the lines were quantitated and exported. Exported data contained the counts along the length of the line in units of pixels. The local maxima of counts for each band in the lane containing the DNA size ladder were used to create a size calibration curve based on the distance along the line drawn through the lane. The calibration curve was fit with a linear equation, which was used to convert length along the line in pixels to estimated size in kilobases of products produced in the rolling circle experiment. The counts along the length of each line were then graphed as a function of estimated length in kilobases to exhibit both product length and abundance in each experimental condition using GraphPad Prism 9.0.

## Data availability

All the data are in the article.

## Supporting information

This article contains supporting information.

## Conflict of interest

The authors declare that they have no conflicts of interest with the contents of this article.

## References

[1] Spelbrink J.N., Li F.Y., Tiranti V., Nikali K., Yuan Q.P., Tariq M. (2001). Human mitochondrial DNA deletions associated with mutations in the gene encoding Twinkle, a phage T7 gene 4-like protein localized in mitochondria. Nat. Genet..

[2] Fratter C., Gorman G.S., Stewart J.D., Buddles M., Smith C., Evans J. (2010). The clinical, histochemical, and molecular spectrum of PEO1 (Twinkle)-linked adPEO. Neurology.

[3] Remtulla S., Emilie Nguyen C.T., Prasad C., Campbell C. (2019). Twinkle-associated mitochondrial DNA depletion. Pediatr. Neurol..

[4] Pierce S.B., Gulsuner S., Stapleton G.A., Walsh T., Lee M.K., Mandell J.B. (2016). Infantile onset spinocerebellar ataxia caused by compound heterozygosity for Twinkle mutations and modeling of Twinkle mutations causing recessive disease. Cold Spring Harb Mol. Case Stud..

[5] Percetti M., Franco G., Monfrini E., Caporali L., Minardi R., La Morgia C. (2022). TWNK in Parkinson's disease: a movement disorder and mitochondrial disease center perspective study. Mov. Disord..

[6] Falkenberg M., Larsson N.G., Gustafsson C.M. (2007). DNA replication and transcription in mammalian mitochondria. Annu. Rev. Biochem..

[7] Sen D., Nandakumar D., Tang G.Q., Patel S.S. (2012). Human mitochondrial DNA helicase TWINKLE is both an unwinding and annealing helicase. J. Biol. Chem..

[8] Korhonen J.A., Pham X.H., Pellegrini M., Falkenberg M. (2004). Reconstitution of a minimal mtDNA replisome *in vitro*. EMBO J..

[9] Fernandez-Millan P., Lazaro M., Cansiz-Arda S., Gerhold J.M., Rajala N., Schmitz C.A. (2015). The hexameric structure of the human mitochondrial replicative helicase Twinkle. Nucleic Acids Res..

[10] Riccio A.A., Bouvette J., Perera L., Longley M.J., Krahn J.M., Williams J.G. (2022). Structural insight and characterization of human Twinkle helicase in mitochondrial disease. Proc. Natl. Acad. Sci. U. S. A..

[11] Korhonen J.A., Gaspari M., Falkenberg M. (2003). TWINKLE Has 5′ -> 3′ DNA helicase activity and is specifically stimulated by mitochondrial single-stranded DNA-binding protein. J. Biol. Chem..

[12] Sen D., Patel G., Patel S.S. (2016). Homologous DNA strand exchange activity of the human mitochondrial DNA helicase TWINKLE. Nucleic Acids Res..

[13] Gao Y., Cui Y., Fox T., Lin S., Wang H., de Val N. (2019). Structures and operating principles of the replisome. Science.

[14] Shutt T.E., Gray M.W. (2006). Twinkle, the mitochondrial replicative DNA helicase, is widespread in the eukaryotic radiation and may also be the mitochondrial DNA primase in most eukaryotes. J. Mol. Evol..

[15] Diray-Arce J., Liu B., Cupp J.D., Hunt T., Nielsen B.L. (2013). The Arabidopsis At1g30680 gene encodes a homologue to the phage T7 gp4 protein that has both DNA primase and DNA helicase activities. BMC Plant Biol..

[16] Peralta-Castro A., Baruch-Torres N., Brieba L.G. (2017). Plant organellar DNA primase-helicase synthesizes RNA primers for organellar DNA polymerases using a unique recognition sequence. Nucleic Acids Res..

[17] Harman A., Barth C. (2018). The Dictyostelium discoideum homologue of Twinkle, Twm1, is a mitochondrial DNA helicase, an active primase and promotes mitochondrial DNA replication. BMC Mol. Biol..

[18] Scherzinger E., Lanka E., Hillenbrand G. (1977). Role of bacteriophage T7 DNA primase in the initiation of DNA strand synthesis. Nucleic Acids Res..

[19] Scherzinger E., Lanka E., Morelli G., Seiffert D., Yuki A. (1977). Bacteriophage-T7-induced DNA-priming protein. A novel enzyme involved in DNA replication. Eur. J. Biochem..

[20] Romano L.J., Richardson C.C. (1979). Requirements for synthesis of ribonucleic acid primers during lagging strand synthesis by the DNA polymerase and gene 4 protein of bacteriophage T7. J. Biol. Chem..

[21] Romano L.J., Richardson C.C. (1979). Characterization of the ribonucleic acid primers and the deoxyribonucleic acid product synthesized by the DNA polymerase and gene 4 protein of bacteriophage T7. J. Biol. Chem..

[22] Kaguni L.S., Oliveira M.T. (2016). Structure, function and evolution of the animal mitochondrial replicative DNA helicase. Crit. Rev. Biochem. Mol. Biol..

[23] Kuhl I., Miranda M., Posse V., Milenkovic D., Mourier A., Siira S.J. (2016). POLRMT regulates the switch between replication primer formation and gene expression of mammalian mtDNA. Sci. Adv..

[24] Fuste J.M., Wanrooij S., Jemt E., Granycome C.E., Cluett T.J., Shi Y. (2010). Mitochondrial RNA polymerase is needed for activation of the origin of light-strand DNA replication. Mol. Cell.

[25] Farge G., Holmlund T., Khvorostova J., Rofougaran R., Hofer A., Falkenberg M. (2008). The N-terminal domain of TWINKLE contributes to single-stranded DNA binding and DNA helicase activities. Nucleic Acids Res..

[26] Peter B., Falkenberg M. (2020). TWINKLE and other human mitochondrial DNA helicases: structure, function and disease. Genes (Basel).

[27] Jumper J., Evans R., Pritzel A., Green T., Figurnov M., Ronneberger O. (2021). Highly accurate protein structure prediction with AlphaFold. Nature.

[28] Itsathitphaisarn O., Wing R.A., Eliason W.K., Wang J., Steitz T.A. (2012). The hexameric helicase DnaB adopts a nonplanar conformation during translocation. Cell.

[29] Peter B., Farge G., Pardo-Hernandez C., Tangefjord S., Falkenberg M. (2019). Structural basis for adPEO-causing mutations in the mitochondrial TWINKLE helicase. Hum. Mol. Genet..

[30] Egelman E.H., Yu X., Wild R., Hingorani M.M., Patel S.S. (1995). Bacteriophage T7 helicase/primase proteins form rings around single-stranded DNA that suggest a general structure for hexameric helicases. Proc. Natl. Acad. Sci. U. S. A..

[31] Jemt E., Farge G., Backstrom S., Holmlund T., Gustafsson C.M., Falkenberg M. (2011). The mitochondrial DNA helicase TWINKLE can assemble on a closed circular template and support initiation of DNA synthesis. Nucleic Acids Res..

[32] Macao B., Uhler J.P., Siibak T., Zhu X., Shi Y., Sheng W. (2015). The exonuclease activity of DNA polymerase gamma is required for ligation during mitochondrial DNA replication. Nat. Commun..

[33] He Q., Shumate C.K., White M.A., Molineux I.J., Yin Y.W. (2013). Exonuclease of human DNA polymerase gamma disengages its strand displacement function. Mitochondrion.

[34] Shadel G.S., Clayton D.A. (1997). Mitochondrial DNA maintenance in vertebrates. Annu. Rev. Biochem..

[35] Miralles Fuste J., Shi Y., Wanrooij S., Zhu X., Jemt E., Persson O. (2014). *In vivo* occupancy of mitochondrial single-stranded DNA binding protein supports the strand displacement mode of DNA replication. PLoS Genet..

[36] Brown T.A., Cecconi C., Tkachuk A.N., Bustamante C., Clayton D.A. (2005). Replication of mitochondrial DNA occurs by strand displacement with alternative light-strand origins, not via a strand-coupled mechanism. Genes Dev..

[37] Falkenberg M., Gustafsson C.M. (2020). Mammalian mitochondrial DNA replication and mechanisms of deletion formation. Crit. Rev. Biochem. Mol. Biol..

[38] Holt I.J., Lorimer H.E., Jacobs H.T. (2000). Coupled leading- and lagging-strand synthesis of mammalian mitochondrial DNA. Cell.

[39] Korhonen J.A., Pande V., Holmlund T., Farge G., Pham X.H., Nilsson L. (2008). Structure-function defects of the TWINKLE linker region in progressive external ophthalmoplegia. J. Mol. Biol..

[40] Nandakumar D., Pandey M., Patel S.S. (2015). Cooperative base pair melting by helicase and polymerase positioned one nucleotide from each other. Elife.

[41] Lo C.Y., Gao Y. (2021). DNA helicase-polymerase coupling in bacteriophage DNA replication. Viruses.

[42] Sun B., Johnson D.S., Patel G., Smith B.Y., Pandey M., Patel S.S. (2011). ATP-induced helicase slippage reveals highly coordinated subunits. Nature.

[43] Patel S.S., Rosenberg A.H., Studier F.W., Johnson K.A. (1992). Large scale purification and biochemical characterization of T7 primase/helicase proteins. Evidence for homodimer and heterodimer formation. J. Biol. Chem..

[44] Matson S.W., Richardson C.C. (1983). DNA-dependent nucleoside 5'-triphosphatase activity of the gene 4 protein of bacteriophage T7. J. Biol. Chem..

[45] Stano N.M., Jeong Y.J., Donmez I., Tummalapalli P., Levin M.K., Patel S.S. (2005). DNA synthesis provides the driving force to accelerate DNA unwinding by a helicase. Nature.

[46] Qian Y., Johnson K.A. (2017). The human mitochondrial single-stranded DNA-binding protein displays distinct kinetics and thermodynamics of DNA binding and exchange. J. Biol. Chem..

[47] Pandey M., Syed S., Donmez I., Patel G., Ha T., Patel S.S. (2009). Coordinating DNA replication by means of priming loop and differential synthesis rate. Nature.

[48] Lee Y.S., Kennedy W.D., Yin Y.W. (2009). Structural insight into processive human mitochondrial DNA synthesis and disease-related polymerase mutations. Cell.

[49] Oliveira M.T., Kaguni L.S. (2010). Functional roles of the N- and C-terminal regions of the human mitochondrial single-stranded DNA-binding protein. PLoS One.

[50] Longley M.J., Smith L.A., Copeland W.C. (2009). Preparation of human mitochondrial single-stranded DNA-binding protein. Methods Mol. Biol..

