# Supplementary material for: The N-terminal domain of human mitochondrial helicase Twinkle has DNA-binding activity crucial for supporting processive DNA synthesis by polymerase γ
Source: J Biol Chem. 2022 Dec 14;299(1):102797. doi: 10.1016/j.jbc.2022.102797 (PMC9860392; doi:10.1016/j.jbc.2022.102797)
Supplement: Supplemental Tables S1–S2 and Figures S1–S4 [file mmc1.pdf]

The N-terminal domain of human mitochondrial helicase Twinkle has DNA-binding activity crucial for supporting processive DNA synthesis by polymerase γ

Laura C. Johnson[1,2], Anupam Singh[1], Smita S. Patel[*1]

[1]Department of Biochemistry and Molecular Biology, Robert Wood Johnson Medical School, Rutgers University, Piscataway, NJ 08854, USA. [2]Graduate School of Biomedical Sciences at the Robert Wood Johnson Medical School of the Rutgers University, USA.

SUPPORTING INFORMATION (FIGURES AND TABLES)

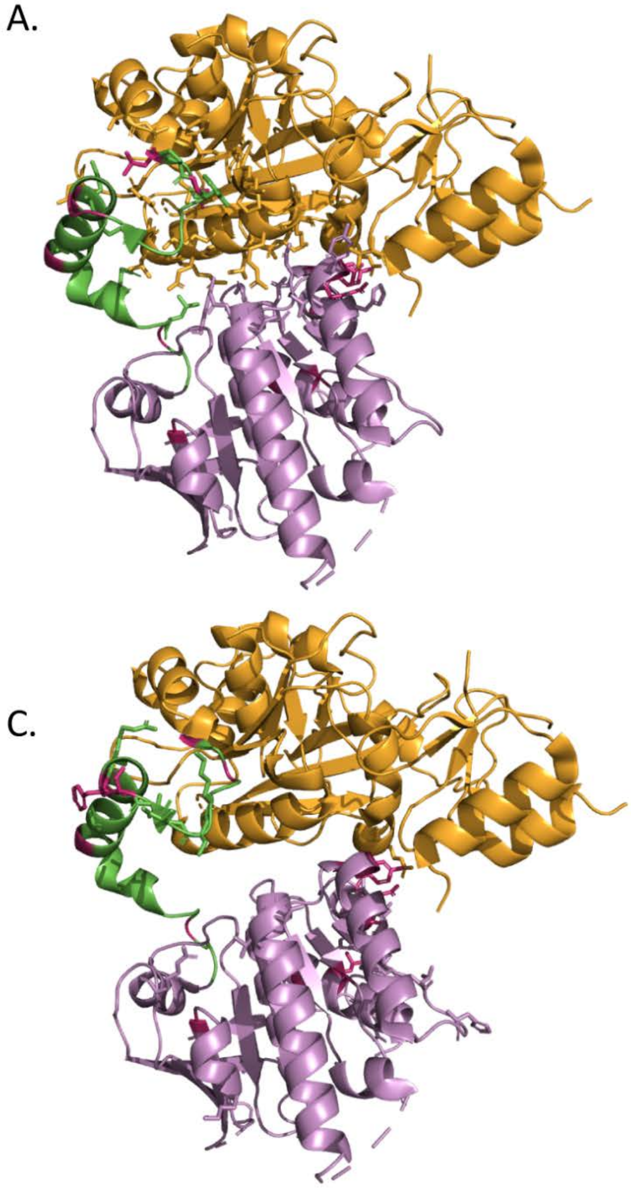

Figure S1 Isolated subunit of the heptameric Twinkle (PDB ID: 7T8C) with residues (sticks) involved in inter- and intra-molecular interactions in Twinkle highlighted within 4 Angstroms and locations of the human clinical disease mutants (in fuchsia). The NTD is displayed in orange, the CTD in purple and the linker in green. **Panels A and B** show different orientations of one subunit of FL Twinkle with NTD-CTD intramolecular interface residues displayed as sticks. The NTD interacts extensively with the linker region involved in subunit interactions. **Panels C and D** show different orientations of one subunit of FL Twinkle with intermolecular interface residues displayed as sticks. Most of the intermolecular interactions between subunits lie in the linker and CTD.

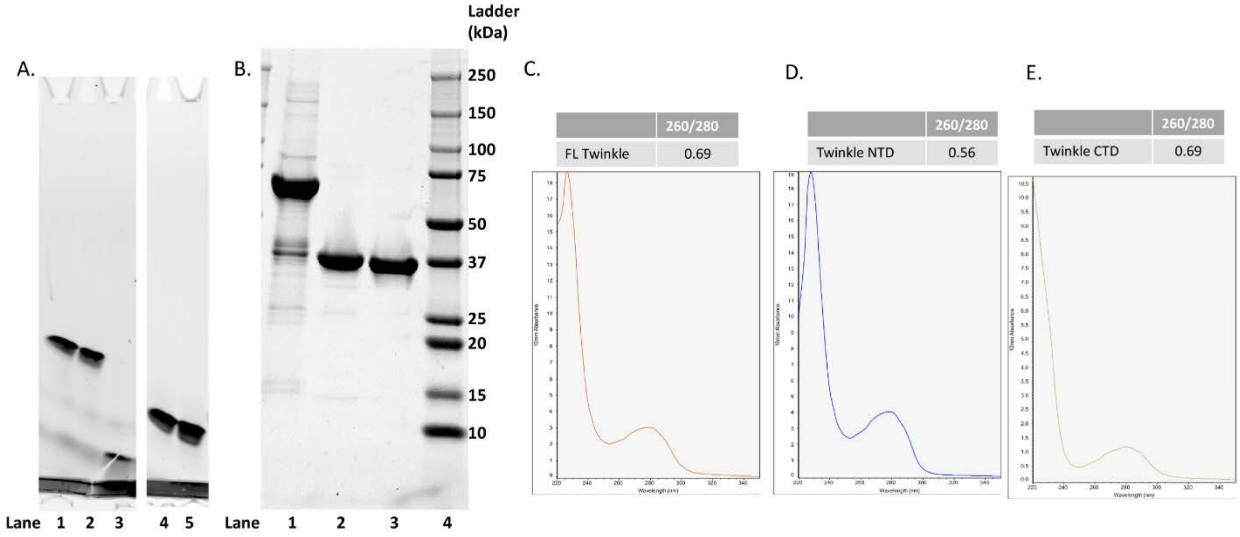

Figure S2 Purified twinkle constructs quality assessment **A.** Gel analysis of nuclease contamination in different Twinkle preparations. 5 nM 35-mer ssDNA labeled with 6-FAM at the 3' end was incubated with 300 nM (hexamer) of insect cell expressed Twinkle purified on Heparin column or Twinkle expressed in *E. coli* and purified with or without Heparin column step. 10 mM $MgCl_2$ was added to the DNA or DNA-Twinkle complexes to initiate the reactions. Reactions were stopped after 30 minutes with 100 mM EDTA and 0.5 % SDS and equal volumes were loaded and analyzed on 4-20 % SDS-PAGE gel. Gel was scanned on FLA Typhoon 9500 scanner (GE Healthcare Bio-Scienses AB, Uppsala, Sweden) to visualize the fluorescent DNA bands. Intact DNA in reactions conducted with Twinkle preparations including Heparin column step confirms that the step removed the contaminating nuclease(s). All the reactions were conducted at 25°C. Lane 1: DNA, Lane 2: DNA + Insect cell expressed FL Twinkle (after Heparin column), Lane 3: DNA + *E. coli* cell expressed FL Twinkle FL (no Heparin column) with strong visible exo activity, Lane 4: DNA, Lane 5: DNA + *E. coli* cell expressed FL Twinkle (after Heparin column). **B.** 4-20 % SDS-PAGE gel of all three purified Twinkle constructs appearing < 95 % pure of other protein contaminants, Lane 1: FL Twinkle, Lane 2: Twinkle CTD, Lane 3: Twinkle NTD, Lane 4: BioRad Precision Plus Protein™ Ladder **C.** Twinkle absorbance spectra and 260/280 ratio **D.** Twinkle CTD absorbance spectra and 260/280 ratio **E.** Twinkle NTD absorbance spectra and 260/280 ratio

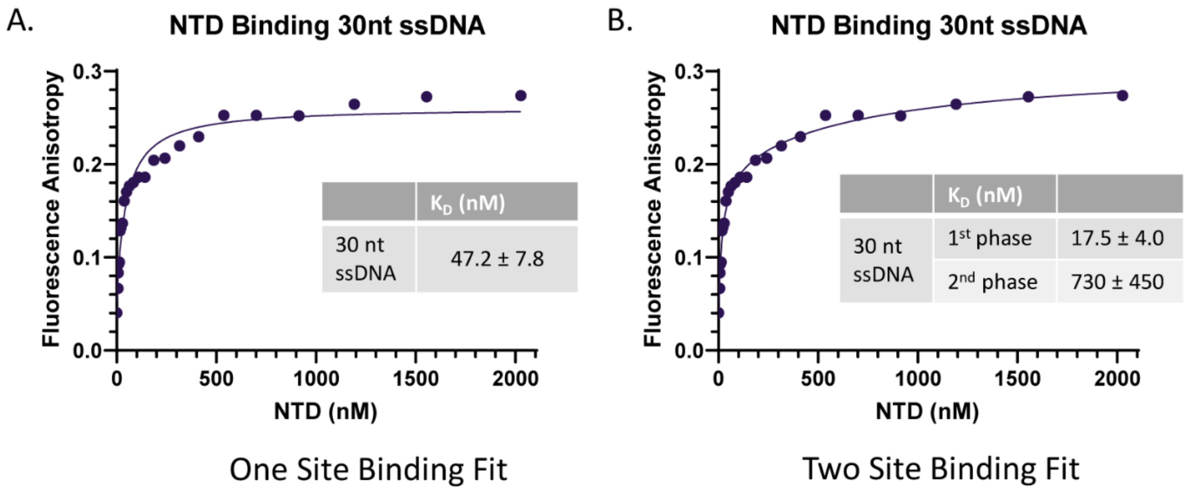

Figure S3 Comparison of binding titration curves of Twinkle NTD binding 30 nt ssDNA fit to either one or two site hyperbolic binding equation, the curve exhibiting clear two phase character suggesting a tight initial binding and a subsequent weaker binding of an additional NTD subunit **A.** one site binding hyperbolic equation $Y = r_{max}*[P]/(K_D + [P]) + Y_0$ **B.** two site binding hyperbolic equation $Y = r_{maxHi}*[P]/(K_{D\ 1st} + [P]) + r_{maxLow}*[P]/(K_{D\ 2nd} + [P]) + Y_0$. Here, r_{max} is the maximum anisotropy, [P] is protein concentration, Y_0 is y-intercept.

Table S1. ssDNA length series constructs used in fluorescence anisotropy binding experiments

ssDNA	Sequence
8 nt	5’ TGAACAC(Fluor T) 3’
12 nt	5’ CTCTTGAACAC(Fluor T) 3’
14 nt	5’ AACTCTTGAACAC(Fluor T) 3’
16 nt	5’ ATAACTCTTGAACAC(Fluor T) 3’
20 nt	5’ TGCTATAACTCTTGAACAC(Fluor T) 3’
30 nt	5’ TGTGTTAGTTGGGGGGTGACTGTTAAAAG(Fluor T) 3’

Table S2. DNA construct components used in short fork strand displacement synthesis, helicase unwinding, and rolling circle synthesis experiments

**Nucleic Acid Assay Components**	**Sequence**
Replication Fork Upper Strand (US)	5' TTTTTTTTTTTTTTTTTTTTTTTTTTTTTTTTTTTTTTTTT TTTTAGCAAGCTCGTACCGTGATCATGCGGCAGGTG TAAGCAC(Invert T) 3'
Replication Fork Lower Strand (LS)	5' (Biotin)AGTGCTTACACCTGCCGCATGATCACGGTAC GAGCTTGCTTTAGGCGAGGTTCGGGACTATCCCTAC TTCCAA(Invert T) 3'
Replication Fork Primer	5' (FAM)ATTGGAAGTAGGGATAGTCCCGAACCTCGC 3'
70 nt Minicircle	- CACCATATCCTCGACCATCCCCAATATGGTCCATCAA CCCTTCACCTCACTTCACTCCACTATACCCTC-
Minicircle Primer	5' TTTTTTTTTTTTTTTTTTTTTTTTTTTTTTTGAGTGGTAT AGTGGAGTGAAGTGAGGTGAAGGGTTGATGGACC ATATTGGGGATGGTCGAGGATATGGT 3'
Unwinding Fork Upper Strand (US)	5' (FluorT)TTTTTTTTTTTTTTTTTTTTTTTTTTTTTTTTTT AGACTTACAATGATCTATACTTAAGAAGATTATCTAT GAACGG 3'
Unwinding Fork Lower Strand (LS)	5' TTCATAGATAATCTTCTTAAGTATAGATCATTGTAAG TCTTTAGCACGCTATGTCGTCAAGTTGTACC 3'
Unwinding Fork Trap	5' TTTTTTTTTTTTTTTTTTTTTTTTTTTTTTTTTTTAGACT TACAATGATCTATACTTAAGAAGATTATCTATGAAC GG 3'

Table S3. Total rolling circle product counts quantified as a proportion of FL+mtSSBs from area under the curve for the product counts over length graphs for each Twinkle construct in the presence and absence of mtSSBs in Figure 8

		**Product (Counts)**
FL	60 min	0.4422
	60 min + mtSSBs	1.000
	60 min + ATPγS	0.02176
	60 min + ATPγS + mtSSBs	0.1005
CTD	60 min	0.01634
	60 min + mtSSBs	0.2048
	60 min + ATPγS	0.01628
	60 min + ATPγS + mtSSBs	0.07977

A.

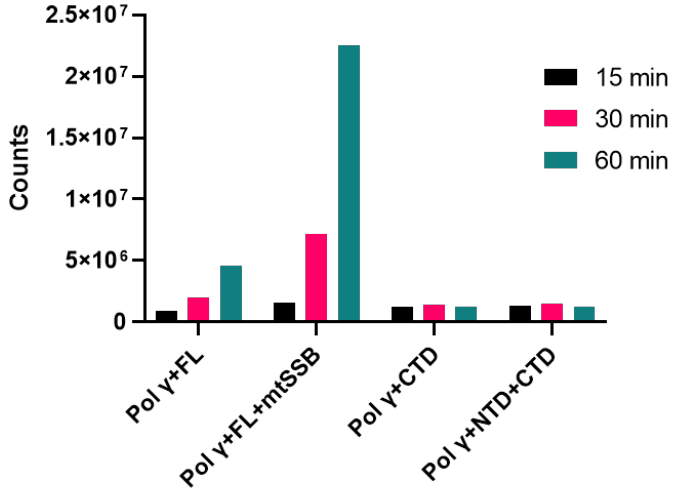

B.

Normalized Product (Counts)	
Pol γ	0.03204
Pol γ + FL + mtSSB	1.000
Pol γ + FL	0.2040
Pol γ + CTD	0.05632
Pol γ + CTD + NTD	0.05505

Figure S4. A. Total rolling circle product count time course from quantitation of the alkaline agarose gel for each Twinkle construct as well as the CTD and NTD acting in trans. **B.** Total rolling circle product counts quantified as a proportion of FL+mtSSBs for each Twinkle construct as well as the CTD and NTD acting in trans.
